# Roll vection in migraine and controls using inertial nulling and certainty estimate techniques

**DOI:** 10.1371/journal.pone.0171332

**Published:** 2017-02-13

**Authors:** Mark Andrew Miller, Benjamin Thomas Crane

**Affiliations:** 1 School of Medicine and Dentistry, University of Rochester, 601 Elmwood Avenue, Rochester, NY, United States of America; 2 Department of Otolaryngology, University of Rochester, Rochester, NY, United States of America; 3 Department of Bioengineering, University of Rochester, Rochester, NY, United States of America; 4 Department of Neuroscience, University of Rochester, Rochester, NY, United States of America; Tokai University, JAPAN

## Abstract

Vection is an illusory perception of self-motion that occurs when a visual motion is presented in the majority of the visual field. We used certainty estimate (CE) and inertial nulling (IN) techniques to study the effect of visual stimuli on roll perception in 10 migraine and 9 control subjects. A visual roll stimulus was presented for 1 to 8s. For the IN method, an inertial stimulus was delivered during the final 1s of the visual stimulus during which subjects judged the direction of perceived motion. The inertial motion was varied to find the point of subjective equality (PSE) at which both responses were equally likely to be reported. For the CE trials, the same durations of visual motion were used but without inertial motion and subjects rated their certainty of motion on a scale of 0–100. The overall difference in PSE between 1s and 8s subjects is significant (p = 0.03). Migraineurs had a ten fold larger effect in IN studies in the 8s than 1s (p = 0.01), but controls did not have a significant difference (p = 0.72). Unlike the control population, in migraineurs the perception of roll increased significantly with the duration of the visual stimulus. There was a large variation between subjects with both the CE and IN measures. The CE measure was poorly correlated with IN measures but demonstrated a similar trend with larger variation between subjects.

## Introduction

Vection is an illusory perception of motion that occurs when a moving visual stimulus is presented in the majority of the visual field [1]. It is important that visual motion be correctly interpreted as vestibular self-motion because the labyrinth cannot always provide reliable information, such as during long, constant velocity motion[2–5]. The sensation of vection is rarely immediate and usually occurs after the stimulus has been present for a period of time on the order of a second when the stimulus accelerates quickly[6, 7].

Several studies have attempted to quantify vection using a variety of techniques. Magnitude estimation is a technique commonly used in vection studies, where the subject assigns a subjective value to their perception [8–13]. This technique is simple to implement and useful in studying individual subjects. Due to the subjective nature of the reporting, however, it is not possible to determine if differences in subjective reporting are due to the underlying perception or due to differences in the interpretation of the stimulus in relation to the reporting method [14–16]. Furthermore, vection magnitude estimation techniques can vary between studies, making study data difficult to compare [8, 13, 17]. Although attempts have been made to calibrate magnitude estimates based on inertial motion [18, 19], this becomes problematic as sequentially presented stimuli may be difficult to match due to adaptation and working memory constraints[20, 21].

An additional technique, direct inertial nulling (IN), is a technique wherein visual and inertial motions are presented simultaneously until the perceived vection is nulled by the inertial force. Vection has been successfully measured in this fashion in fore-aft, lateral, and rotational studies [14, 22–24]. While these studies have demonstrated the feasibility of the technique, the task used in some previous studies would be difficult in clinical populations. For instance, continuously moving a pointer to null perception was complex enough that only a minority of young healthy subjects were able to reach criterion [25].

Roll rotation presents some interesting challenges for human motion perception. The feeling of vection (i.e. self motion induced by a visual stimulus) is largest with visual stimuli that fill a large part of the field of view[26], at very low rotation frequencies (below 0.06 Hz)[27, 28], and high visual spatial frequencies[29, 30]. What is less clear from the prior literature is the origin of intra-subject variation in roll rotationi despite significant variation being present in vection studies [13, 31]. Bubka et al. postulated that recent history may modify vection experience, and the adaptation to visual stimuli[8]. In addition to variation in the reporting scale being a limitation of magnitude estimation techniques [15, 16], migraine diagnosis may be another factor. Migraine is extremely common in the healthy population occurring in 18–24% of women [32, 33]and a diagnosis of migraine may be an important confounding variable in vection studies and explain some of the variability in vection experience as previously reported for for-aft vection [34].

Some recent results indicate roll-tilt vestibular perception is abnormal in subjects with vestibular migraine [35]. This study demonstrated that the threshold of low frequency tilt was lower in subjects with vestibular migraine relative to controls, a finding the authors attribute to abnormal multisensory (in this case semicircular canal-otolith) integration. Furthermore, migraine has been shown to influence visual perception with prolonged visual motion after effects[36–38], increased thresholds of visual motion detection[39], and aversion to visual patterns[40]. Given these findings it seems reasonable to investigate the possibility of visual-vestibular integration also influencing roll/tilt perception in migraineurs. It is especially relevant considering feelings of tilting frequently occur during migraine episodes [41–45] and some subjects with a migraine history have a stronger perception of vection [34].

The current study reports the magnitude of vection in the roll plane using IN and in a magnitude estimation technique refereed to as certainty estimation (CE) in which subjects report the probability a stimulus represents self motion in healthy controls and those with migraine.

## Methods

### Ethics statement

Written informed consent was obtained from all participants. The protocol including the written consent document was approved by the University of Rochester Research Science Review Board and conducted according to the principles expressed in the Declaration of Helsinki.

### Subjects

We recruited 10 subjects who met clinical criteria for the diagnosis of migraine (9F, 1M) and 9 control subjects (6M, 3F) with no known history of visual or vestibular symptoms. All migraineurs met International Headache Society criteria for migraine (The International Classification of Headache Disorders 2004). None of the migraine subjects met criteria for vestibular migraine[46]. All subjects were right handed. The mean age for migraineurs was 31 (SD = 15.6). All migraineurs were white. The mean age for controls was 23 (SD = 2.9). Five controls were white and 4 were Asian. All subjects underwent general screening for history of dizziness, vertigo, hearing and vision problems. History of neurologic problems, and rheumatic disease was also explored. All subjects were right handed.

### Equipment

The motion used in the study was delivered using a six-degrees of freedom Hexapod Motion Platform (HMP) (Moog, East Aurora, NY, USA, model 6DOF2000E) with an attached visual display. This allowed inertial motion to be synchronized with a visual stimulus as previously described in this laboratory [34, 47, 48].

During the experiments, subjects were seated in a padded racing style seat which was mounted to the platform (Corbeau, Sandy UT, model FX-1). Helmets fixed to the HMP were available in several appropriate sizes ensuring that head and platform movements were closely coupled.

Masking noise from two platform-mounted speakers was used as previously described[49]. The intensity of the white noise was varied during both platform and visual motion so that peak masking occurred at the time of peak motor noise which did not depend on the magnitude of the stimulus, although it was also present in visual only trials for consistency. The intensity of the noise was independent of the direction of platform motion and was delivered for both inertial nulling, in which platform motion occurred, and certainty estimate trials, wherein platform remained stationary.

A two-dimensional computer generated image consisting of white circles that simulated roll in a star-field by rotating about a central point [50] was used. The stimulus was presented on a LCD screen which filled 98° of the horizontal field of view, had a standard 16:9 (horizontal:vertical) aspect ratio, and the viewing distance was 25 cm. Each star consisted of a circle with a diameter of 0.05 cm in the plane of the screen. The star density was 0.002 per cubic cm. Except for the light from the screen, conditions were performed in darkness. Blinders around the edges the screen masked the surrounding walls to avoid potential visual cues to platform motion. The method by which the timing of the visual and inertial stimuli were synchronized has previously been described for the current laboratory[47]. No fixation point was used.

Following platform and visual motion, subjects were instructed to indicate the perceived direction of motion (either clockwise or counterclockwise) by pressing the appropriate button with a hand-held button box, making this a single interval, forced choice task. After the response was reported, the platform returned to the starting position.

### Experimental procedures

Study blocks were broken into two to three sessions, each lasting between one and three hours. Trial blocks were randomly ordered between subjects. When participating in the experiment, all subjects were offered breaks between trial blocks to prevent fatigue.

#### Inertial nulling trials

Control trials were performed to determine baseline bias in visual and motion perception. Baseline inertial bias (i.e. shift of the mean of the psychometric function relative to zero) was measured in the *platform motion control* trial, with platform motion in darkness (no visual motion counterpart). Baseline visual bias was measured in two separate trials: platform motion with a *static visual stimulus* and platform motion with a *zero coherence visual stimulus*. For each of these trials, a 1s visual stimulus was presented simultaneously with a 1s inertial motion stimulus, and the subject was instructed to indicate the direction of perceived self-motion during the inertial stimulus which was marked by an audible cue. If they did not perceive any motion they were instructed to guess its direction to the best of their ability. The stimulus was presented to the subject 36 separate times in each control trial.

Platform motion was delivered during the final 1s of PSE blocks, and consisted of a sine wave in acceleration with a maximum velocity of 8 degrees/sec delivered in the roll plane. The motion was free of discontinuities in acceleration, velocity, or position as previously described[49].

Three separate trials were conducted with visual stimulus durations of 1, 4, or 8s. In these trials, the visual star field rotated at a constant 55 degrees/sec in either clockwise or counterclockwise directions, and an inertial motion stimulus was delivered by platform motion during the final 1s of the visual stimulus in the clockwise or counterclockwise direction. Blocks of motion perception trials were presented in random order to the degree possible. The short duration (1s) stimulus was chosen as a control when no vection was likely to occur as roll vection has previously been shown to require a longer duration stimulus[7]. It was felt that if an effect did occur at 1s it would be likely due to a visual-vestibular integration phenomena rather than vection. It was recognized that longer duration stimuli that those tested (8s) might have been a more compelling vection stimulus. Simuli longer than 8s were not included for two reasons. First because each trial block included 72 stimulus presentations making the stimulus longer also made an already long trial block longer. Trial blocks longer than about 30 minutes caused issues with some subjects being able to maintain attention. Second, we wanted to include migraineurs in this study and some migraineurs would experience motion sickness symptoms after longer visual stimuli which would limit their participation in the study.

An adaptive staircase was used to determine the point of subjective equality (PSE), i.e. the point at which subjects were equally likely to perceive motion in either direction. The staircase used a one-up, one-down variable step size. Each block was designed to start with a large platform motion stimulus that was likely to be unambiguously perceived, with the stimuli becoming incrementally smaller as the experiment progressed. Two sets of staircases where used, these sets were randomly interleaved with each set representing opposite directions of rotation. This was done to limit the effects of adaptation to one direction of motion. Within each set, two independent staircases were used for platform (inertial) motion. One staircase started with an 8 deg/sec clockwise platform motion, and one started with an 8 deg/sec counterclockwise platform motion. Thus, each trial block included 4 randomly interleaved staircases with 18 stimulus presentations per staircase (72 total). Staircases were randomly interleaved to decrease the ability of subjects to predict stimuli presentation based on prior experience.

#### Certainty Estimation (CE) trials

Certainty Estimation (CE) trials were conducted in a similar fashion as the IN trials, but without platform motion. The same roll visual stimuli were presented at the same speed. Three control blocks and three visual field motion (VFM) blocks (1s, 4s, and 8s) were conducted, with each block consisting of 4 stimulus presentations. Subjects were instructed to verbally report perceived direction and certainty of self-motion at the conclusion of the stimulus based on a scale of 0 to 100, a scale that has been used successfully in previous work aiming to quantify vection [13, 51, 52]. Zero was defined as no perception of motion, and 100 defined as “extremely compelling”, and no additional reference points were suggested as subjects have been found to replace these with their own internal reference values [15]. We chose to use certainty rather than confidence as the measure to maintain consistency with some prior work in this area[34], but this also better fits the proposed definition of certainty because subjects were not actually asked to decide of the motion represented self motion or external motion[53]. In these trials subjects were not asked to report the direction of motion as the visual motion was large enough that the motion relative to the observer was unambiguous. The only ambiguity was if this motion represented environmental motion relative to a fixed observe or observer motion through a fixed environment.

### Data analysis

**Fig 1:**
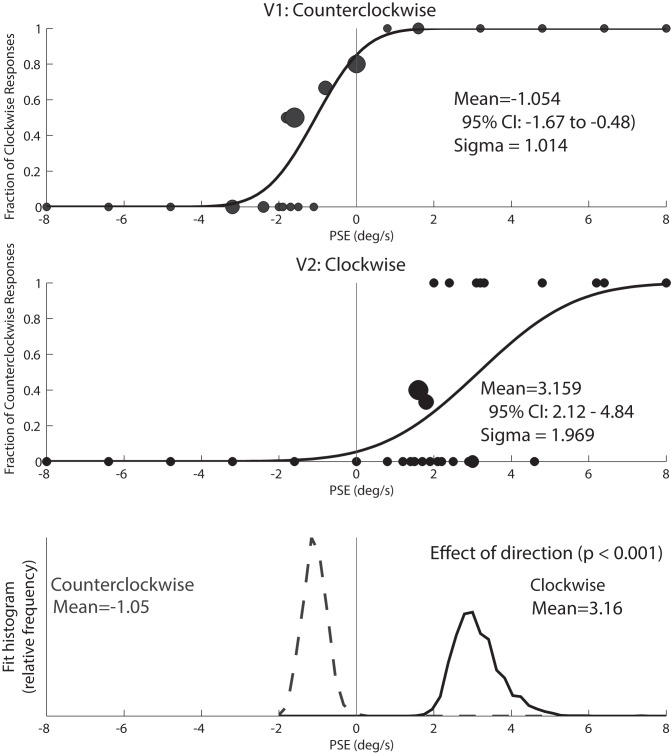
Individual psychometric fitting for an 8s PSE stimulus in an individual subject. Circles in the two upper panels are sized proportionally to the number of responses represented. A cumulative distribution function (CDF) was calculated from each data set as a method for determining the mean (bias) and sigma (threshold) of inertial motion detection for each test condition. Each panel combines data from 2 staircases that started at opposite extremes (-8 and +8 deg/s). Both staircases could cross zero depending on the subject’s responses. In this subject, the CDF had a significant shift towards the right when clockwise motion was presented (top panel) when compared with counterclockwise visual motion (middle panel). Each CDF was fit to the data 2,000x after being randomly resampled prior to each fit. The histograms of these fits are shown in the bottom panel which demonstrates a significant difference between the two conditions based on no overlap between the curves. The area under each curve is the same, and the y axis is arbitrary. The counterclockwise stimulus is represented by dashed line, clockwise stimulus is represented by the solid line.

For the IN trials, the proportion of clockwise to counterclockwise responses was modeled by a cumulative Gaussian function using a Monte Carlo maximum-likelihood criteria as previously described and used in the current laboratory. Data were resampled randomly with replacement to generate multiple estimates of the mean and 95% confidence intervals [54, 55]. Psychometric fitting for a typical subject is shown in Fig 1. The point of subjective equality was defined as the mean of the Gaussian distribution, and represents the motion force that elicits responses divided equally between the two possible responses. Deviations from a mean of zero represent an inertial nulling force that is equal and opposite to the perceived direction of motion (vection). For analysis purposes, effects in opposite directions during IN trials could be combined by subtracting the PSE with counterclockwise visual field motion (VFM) from the PSE with clockwise VFM and dividing by two. Threshold was defined as the sigma or width of the cumulative Gaussian distribution, and the level of significance in the difference of the means of clockwise vs. counterclockwise was defined as p < 0.05 as in previous studies [47].

Two-way ANOVA with repeated measures was used for comparisons of population (two levels: control or migraine) and duration (three levels: 1, 4, and 8s). Pearson’s correlation coefficient was used to test correlation between the PSE and CE trials, and between clockwise and counterclockwise tests within each trial. Statistical significance was defined as p < 0.05.

## Results

### Inertial Nulling (IN)

The experiment was well tolerated and all subjects completed all test conditions. All subjects were able to correctly identify the direction of the inertial stimulus at the start of the staircase (the platform motion stimulus of largest magnitude).

**Fig 2:**
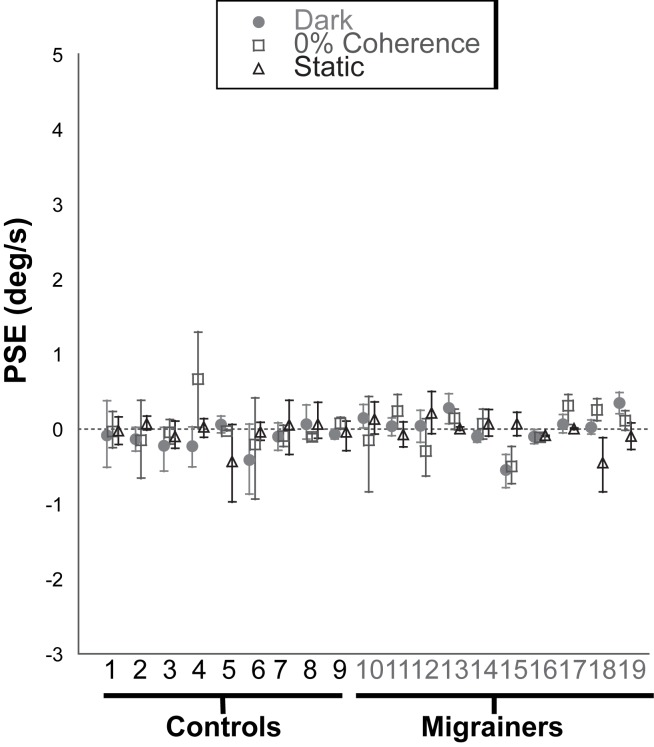
Individual Subject Data for Control Trials. Platform motion in darkness is demarcated by filled circles, platform motion with a 0% coherence visual stimulus is shown with open squares, and platform motion with a static visual stimulus is shown with triangles. Error bars represent 95% confidence intervals. The y axis limits were set to be consistent with Fig 3.

Baseline inertial bias (measured with the *platform motion control* trial), as well as baseline visual bias (measured with *static visual stimulus* and *0% coherence trials)*, was minimal (Fig 2). Small biases were noted in several subjects but all were under 1 degree/s, and no subject was excluded based on these initial findings.

**Fig 3:**
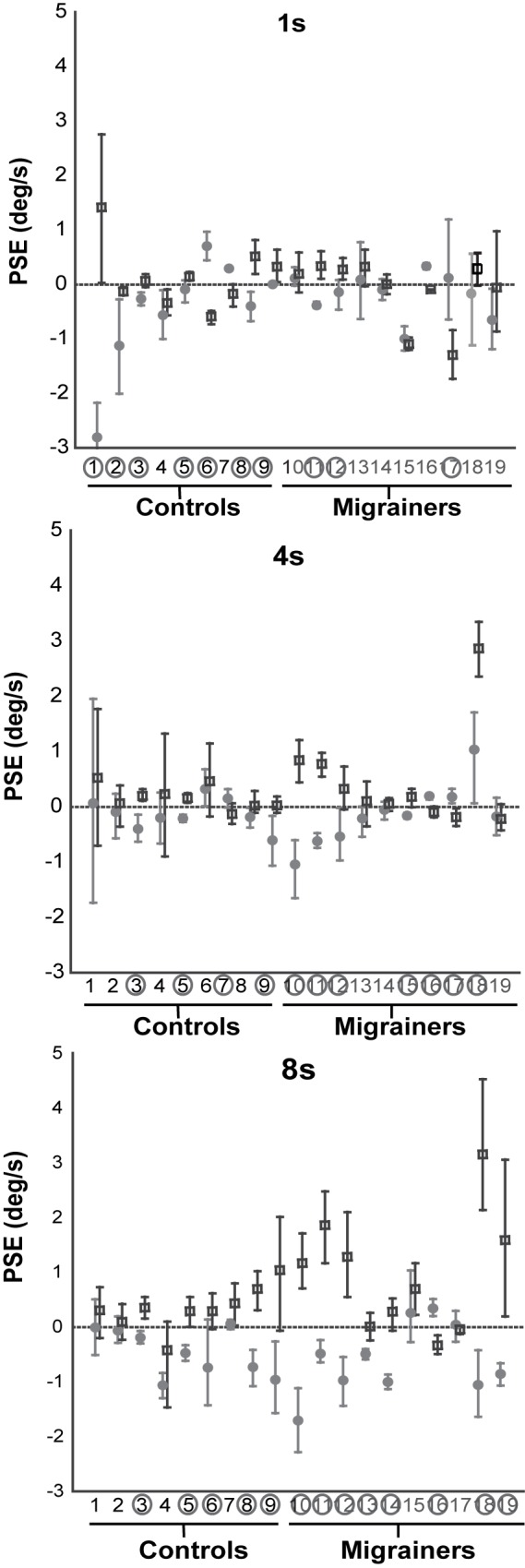
Individual subject data for 1s, 4s, and 8s PSE trials. Counterclockwise visual field motion (VFM) represented with filled circles, clockwise VFM with open squares. A positive PSE indicates that a neutral motion would be more likely to be perceived as clockwise self-motion, likewise a negative PSE indicates a neutral motion would be perceived as counterclockwise. Error bars represent 95% CI.

**Fig 4:**
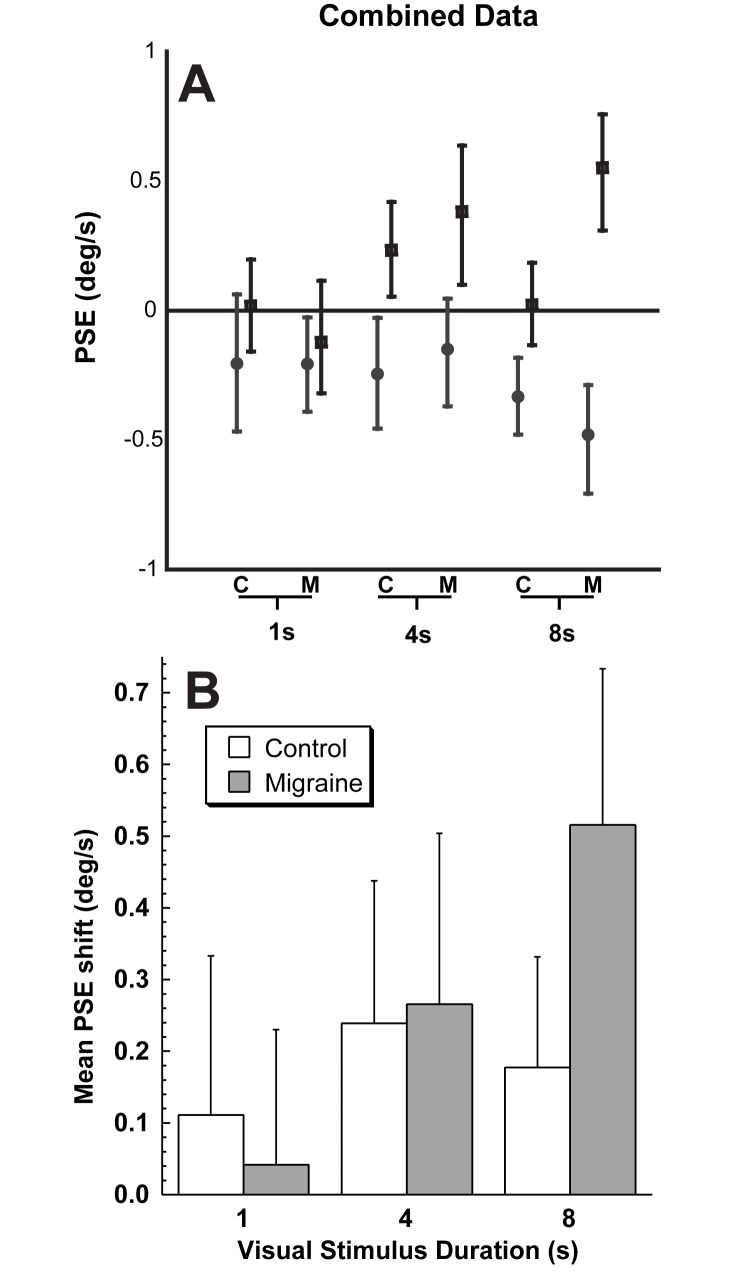
Combined subject data by population and trial. Panel A: Counterclockwise VFM is represented with filled circles, clockwise VFM with open squares. A positive PSE indicates that a neutral motion would be more likely to be perceived as clockwise self-motion, likewise a negative PSE indicates a neutral motion would be perceived as counterclockwise. Error bars represent 95% CI. Panel B: Both directions are combined to show the mean shift. Control subjects are represented by open bars and migraineurs by filled bars.

**Fig 5:**
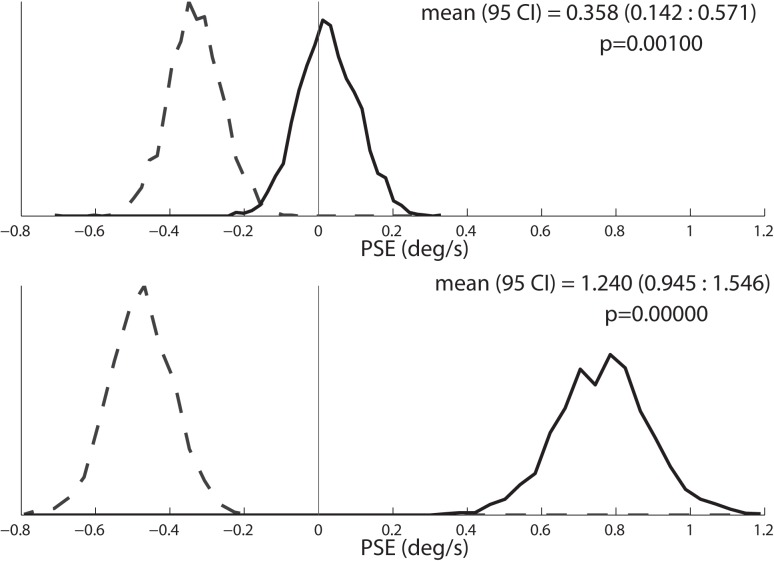
Histogram comparing combined controls and migraineurs response at 8s using a random resampling of responses. Responses from all subjects were included. Responses collected with counterclockwise VFM are represented with a dashed line, clockwise VFM responses are represented with a solid line. The area under the curve in both plots represents the total number of fits (2,000), no specific units are given for the y axis.

Results for individual IN trials based on duration are shown in Fig 3, and combined data are shown in Figs 4 and 5. For IN trials, the difference in PSE between 1s and 8s in all subjects was significant (t-test, p = 0.03) indicating a significantly greater effect in the longer duration trials. When examined by population, migraineurs had a greater shift in PSE in IN studies in the 8s than 1s (t-test, p = 0.01), but controls did not (p = 0.72). For migrainers the PSEs at 8s were 0.55 deg/s for clockwise VFM vs -0.49 deg/s for counterclockwise VFM. The corresponding PSEs at 1 s were -0.12 and -0.21 deg/s. Thus at 8s the PSE difference between clockwise and counterclockwise VFM was 1.04 deg/s and for 1s the corresponding difference was 0.09 deg/s. Thus the effect of VFM on PSE was an order of magnitude larger when the 8s stimulus was compared with the 1s stimulus.

Using a two-way ANOVA by population (control/migraine) and stimulus duration, the effect of stimulus duration was highly significant (p<0.0001) and the variation between individual subjects was significant (p = 0.0008). However when all time points were considered was no significant difference between the control and migraine population (p = 0.55) and the interaction between the subject group and time was not significant (p = 0.32)

### Certainty Estimation (CE)

Baseline inertial bias (i.e. shift of the PSE from zero as measured with the *platform motion control* trial), as well as baseline visual bias (measured with *static visual stimulus* and *0% coherence trials)* were performed, and no subject had a deviation >1 (on a 0–100 scale).

**Fig 6:**
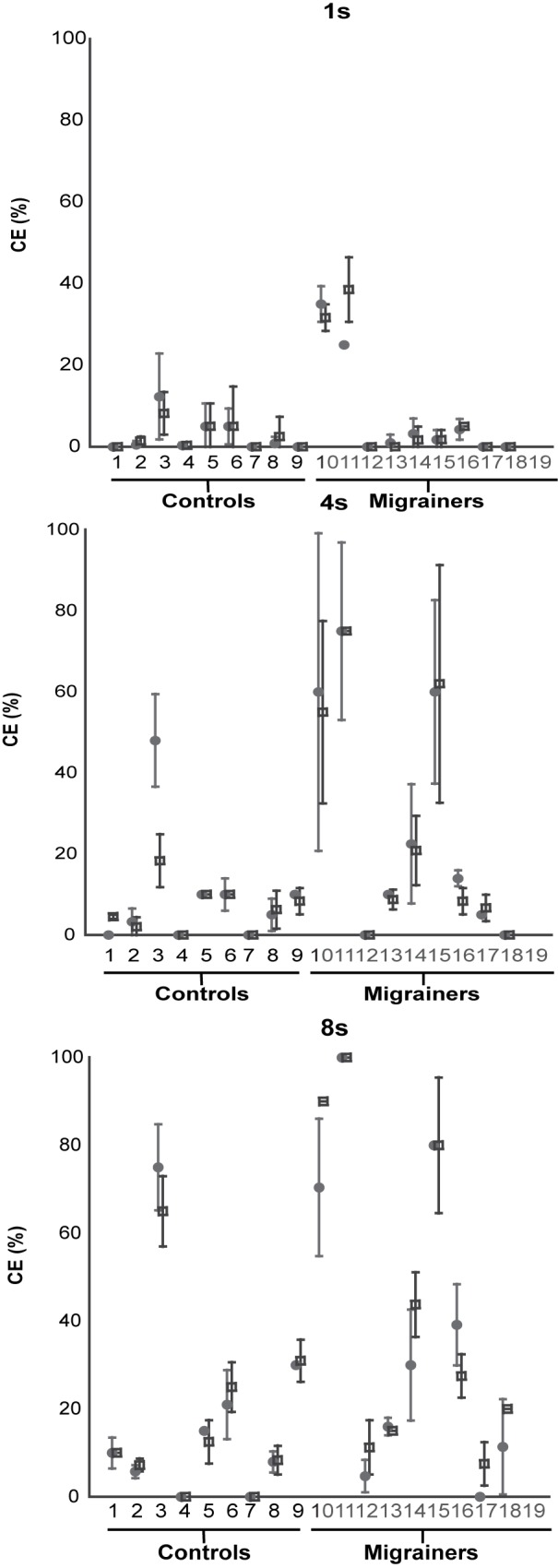
Individual Subject Data for 1s, 4s, and 8s CE trials. Three graphs represent individual subject means for 1s, 4s, and 8s trials, respectively. 8 stimuli were delivered in each trial as either the clockwise or counterclockwise VFM. Mean certainty estimates for each subject were collected with counterclockwise VFM represented with a solid circle, clockwise VFM responses are represented with a square. Error bars represent 95% CI.

Subject-reported certainty estimates of self-motion for visual motion trials are shown in Fig 6. Migraineurs had a significant difference between 1s and 8s trials (student t-test, p = 0.03 in CW, p = 0.02 CCW), but controls did not quite reach significance (p = 0.08 in CW, p = 0.06 CCW). Two-way ANOVA by population and duration for both CW and CCW trials was performed. There was a significant difference in results based on duration (1s, 4s, and 8s) in both CW and CCW trials (p<0.01), and individual subjects (p<0.01), but not based on population alone (CW, p = 0.12; CCW, p = 0.19). The interaction between population and duration was not significant (CW, p = 0.24; CCW, p = 0.10).

### Inertial nulling and certainty estimate correlation

The correlation between CE and IN was small to modest across all trials. Interestingly, the correlation between clockwise and counterclockwise studies was modest in all IN trials (8s trial, r = 0.44), whereas the correlation for CE was unusually high (8s trial, r = 0.97). The variance among migraine subjects at the 8s (σ^2^ = 2.32) was much greater than controls (σ^2^ = 0.35) in IN trials.

## Discussion

The current study examined vection in the roll plane using certainty estimates (CE) and inertial nulling (IN) techniques. The major findings of the current study demonstrated was that vection perception with the IN technique increased significantly (p < 0.01) by about ten fold in migraineurs with stimulus duration a trend not present in controls (p = 0.72; Fig 4). A similar but noisier trend was also seen when vection was measured using the CE technique (Fig 6). Vection as measured with the IN and CE techniques were poorly correlated and there were large variations between subjects even within the migraine and control groups. Previous studies in this laboratory used the same IN tool to test vection in control and migraine subjects in the fore-aft plane, demonstrating a good correlation between inertial nulling and certainty estimate in the longer duration trials where vection would be experienced[34]. The roll vection effect was seen in 14 of 19 subjects in the current study with an 8s stimulus but in 8 of 18 subjects with a similar fore-aft stimulus[34] which suggests roll produces a more compelling or universal sensation of vection. The current data are consistent with studies that have shown migraineurs are more likely to demonstrate larger variations in postural sway and visual vertical perception with rotating visual stimuli[56].

The vection phenomenon is well known, and results in most subjects interpreting that the visual motion as representing their own self motion through a fixed environment. However, it is possible that some subjects would interpret vection as a motion in the same direction as the visual motion, perhaps as if the visual objects were pushing them in the same direction but at a lower rate of speed. Our IN technique allowed perception of motion in either direction to be quantified. Using a Monte Carlo technique with resampling of data points we were able to determine significant effects for individual subjects and in one subject (#16) this opposite perception was significant with an 8s and 4s visual stimulus suggesting it was not just a result of chance. Furthermore this subject demonstrated the same effect, although not significant using the Monte Carlo technique with the 1s visual stimulus. All subjects were given the same instructions and it is difficult to attribute this effect to a simple misunderstanding of the task. For instance reporting only the direction of the visual stimulus without regard to the inertial stimulus would have created a set of responses that could only be fit to a psychometric function only if the mean of the function was well outside the range tested, which was not what was observed. The perception in this subject may have represented an unusual migraine variant. Perhaps, an argument could be made to remove this subject which would have resulted in a larger vection effect in the migraine subjects but would not have qualitatively changed the aggregate findings.

There are multiple reasons why roll vection perception in migraineurs may have significantly increased with stimulus duration. Enhanced visual cortex excitability has been shown in migraineurs[57, 58] although it is not clear that this is the reason for increased sensitivity to motion in this population[40]. Thus, the visual stimulus may be over-represented in the visual cortex and transfer to a sense of roll. During real world conditions roll is usually associated with both a visual and vestibular stimulus, which were decoupled from each other in the current experiments. Another possibility is that effect may be due to an abnormality with visual-vestibular multisensory integration in which stimulus of one sensory modality can spill over into other sensory modalities more readily in migraineurs[59]. However, neither of these theories explains the observed dependence on the duration of visual motion in migraineurs. Prolonged visual motion after effects have been shown in patients with migraine[36–38], and these motion after effects are dependent on the stimulus duration. Thus a visual motion aftereffect etiology may be more feasible. It was interesting that there was no significant effect of stimulus duration on vection in the control group. It is possible that significant vection effects could have been measured in the control group using different stimulus parameters such as a longer visual stimulus. Use of a significantly longer stimulus was not possible using the current techniques, thus it is possible the vection effect seen in the migraine population with the 8s stimulus may also be seen in controls using a longer duration stimulus.

It is known that vection is not immediately perceived with a visual stimulus but is often delayed[6, 7]. The latency of vection with a roll stimulus was has been previously been examined and is found to be a function of the acceleration of the visual stimulus[7]. At low accelerations such as 0.1 deg/s/s the latency of vection is longer and on the order of 10s. This decreases to the order of 1s with stimuli at accelerate at 1 deg/s/s or faster. In the current study the acceleration of the visual stimulus was not examined and a constant velocity (55 deg/s) stimulus was used which would correspond with a high acceleration stimulus. The current data demonstrated a minimal effect of vection in most subjects at 1s with larger effects at 4 and 8s consistent with the latency of vection being about 1s. Of course it is possible that the intensity of vection continues to increase with time. Such an effect seems to be present in subject #9 (Figs 3 & 6) when comparing 4 and 8s but was not generally the case in controls (Fig 4) although it was seen more consistently in migraineurs.

There were limitations to our current study. Migraine is a broad diagnosis encompassing different symptoms and severity, and population heterogeneity may account for some of the variation in vection. The control group had younger subjects (mean age 23) than the migraine ground (mean age 31). Prior work in our lab that looked at age effects found no significant effect of age with age < 50. Above age 50, there is a lot more variation but some individuals have decreased performance[48, 49]. Another recent study found similar results but conservatively put the age cut off at 40[60]. Although our migraine group was predominately female consistent with the female predominance of migraine in the population, to the authors’ knowledge no previous study has found a significant difference in vestibular perception with gender even though the issue has been examined by several groups[49, 61, 62]. Previously studies on inertial nulling have also not found gender influences[34], although it is not clear as some studies never reported on the issue[18], and other study included almost all males[14]. However, the potential effect of gender in the current paradigm has not been previously studied, and there is a small possibility that gender rather than migraine history may be responsible for some of the effect seen. Female gender is known to be a major risk factor of migraine[33], so it may not be completely possible to disentangle gender effects from migraine effects in a population. Studying cross modal aftereffect with visual and inertial translation stimuli, found that sustained vection with a stimulus of 15s was necessary to elicit cross modal transfer[63] in normal human subjects. However, our previous study didn’t show enhanced vection from 8s to 16s, and the current study demonstrated a more robust vection effect with 8s of roll stimulus than the comparative 8s fore-aft stimulus of the previous study[34].These prior studies did not examine roll, but it is possible that if 15s+ stimuli were used the duration effects seen in migraineurs might have also been seen in the control population study and there may have been stronger effects in both groups. Because of the range of motion possible with the current apparatus 15 s stimuli would be impractical to test. In addition, in our preliminary studies we noticed that some migraineurs got motion sickness symptoms with longer visual stimuli which impaired their ability to complete the study. Thus it might be difficult to use longer stimuli in a study that included migraineurs without biasing the study population. Thus it is possible that the current findings represent a phenomenon that occurs at shorter latency in migraineurs relative to controls.

## Conclusions

Our data demonstrate the use of inertial nulling technique in measuring vection in the roll plane, and the differences between CE and IN. Longer stimulus durations led to enhanced vection in migraine to a greater degree than see in the control subjects in the roll plane. However, there was a large variation between subjects in both the control and migraine group suggesting other factors may have had a significant influence on vection perception.
